# Is agritourism eco-friendly? A comparison between agritourisms and other farms in Italy using farm accountancy data network dataset

**DOI:** 10.1186/s40064-015-1353-4

**Published:** 2015-10-12

**Authors:** Luigi Mastronardi, Vincenzo Giaccio, Agostino Giannelli, Alfonso Scardera

**Affiliations:** Department of Economics, Management, Society and Institutions, University of Molise, Campobasso, Italy; National Institute of Agricultural Economics (INEA), Campobasso, Italy

**Keywords:** Agritourism, Farm, Environmental performances, Italy

## Abstract

This paper presents the results of research regarding the environmental performances of Italian farms with agritourism compared with farms without agritourism. In Italy, agritourism is considered an agricultural activity and can only be performed by a farmer. Moreover, Italian national legislation forces the farmer to dedicate himself mainly to traditional farming, rather than to tourism activities. For this reason, environmental performances have been highlighted by analyzing only features and production systems of the farms. By utilizing the most frequent indicators used in studies regarding sustainability, the authors show how Italian agritourisms tend to develop more environmentally friendly agricultural methods, which have a positive impact on biodiversity, landscape and natural resources. The empirical analysis is based on the Italian FADN (Farm Accountancy Data Network) dataset. The European FADN was created to represent farms’ technical and economic operation in the European Union and on which it drafts the agricultural and rural policies. The dichotomous structure of the dependent variable (presence or absence of agritourism at the farm) has a propensity for an assessment method based on Binary Response Model Regression.

**Electronic supplementary material:**

The online version of this article (doi:10.1186/s40064-015-1353-4) contains supplementary material, which is available to authorized users.

## Background

This paper analyses the connection between agritourism and environment, by comparing the environmental performances of agritourisms with non-agritourism farms. It aims at realising if agritourism has a low environmental impact in the Italian agricultural system.

Agritourism is considered a subset of rural tourism (Phillip et al. 2010) which is based on the use of the resources present in the countryside (Cawley and Gillmor 2008; Hall et al. 2003; Roberts and Hall 2001) and finds its basis in the new models of consumption (Amin 1994) and enjoyment of rural areas (Ray 2003).

In any case, rural tourism is a complex and vastly differentiated phenomenon (Frochot 2005) the effect of which depends on the characteristics of each individual territory and the manner in which the public and the private actors set up the relationships between tourism products and local resources (Pacciani 2011).

In literature, the meaning of agritourism is rather controversial (Colton and Bissix 2005; Guerrero Velasco et al. 2012; Lane 1994) and, consequentially, its aims seem unclear, as does the relationship that ties this activity to working farms.

This relationship often refers to farms as physical rather than financial entities in which agriculture and/or livestock farming are regularly practiced (Kizos and Iosifides 2007; Sonnino 2004; Tew and Barbieri 2012).

In every case, literature is unanimous in considering agritourism as a key factor for local development (Slee et al. 1997; EC 2007; Saxena et al. 2007), in particular for rural marginal areas (Dimara and Skuras 1999), or in areas where the environmental and cultural heritage are strongly appreciated by tourists (Garrod et al. 2006).

Even though there is a growing interest in the economic and social benefits of agritourism (Koutsouris et al. 2014; McGehee et al. 2007; Tew and Barbieri 2012; Vogt 2013), literature presents significant gaps in terms of its environmental consequences (Nickerson et al. 2001; Oppermann 1995; Veeck et al. 2006). As regards the environmental performances, empirical analysis, mostly case studies, highlight that agritourism can produce both positive and negative effects on the environment and on the socio-economic context in which it is developed (Daugstad et al. 2002; Frey and Zimmermann 2005). For instance, it is associated with positive effects on some environmental components, such as landscape, water and energy resources, biodiversity, as well as reduced use of fertilizers and pesticides in productive processes and improvement of the quality of foods (Giaccio and Mastronardi 2011; Mastronardi et al. 2015). This occurs when the touristic demand pays close attention to the principles of sustainable performances, to quality products and to those which are of low environmental impact (Negri 2005). The demand made by tourists for traditional agricultural landscapes is continually increasing (Daugstad 2008; Soliva et al. 2008; Walford 2001), as it is for those agricultural landscapes that are heterogeneous (Daugstad et al. 2006) or perceived as a source of mental, physical and spiritual wellness (MacDonald and Jolliffe 2003). As to this, it is useful to recall that farming has a very important role in the conservation of natural resources and in landscape conservation. Agriculture produces positive external effects (Non-Commodity Outputs—NCOs) (OECD 2001a, 2001b, 1998; Van Der Ploeg and Roep 2003; van Huylenbroeck et al. 2007), which bring a clear advantage on a social level (Abler 2003; Deuffic and Candau 2006).

On a normative level, the EU makes generic reference to agritourism as a form of holiday, which is carried out in rural areas. Most EU countries, however, equate agritourism with other forms of rural tourism (Marcotte et al. 2006) and this has produced a limited increase in the phenomenon in the UE (Fleischer and Pizam 1997; Oppermann 1996; Vogt 2013), particularly in areas with a long tradition of rural tourism, as well as in areas with scarce involvement of farms and, consequentially, mainly tourism firms in rural environments that do not carry out any agriculture.

Italian legislation regulates agritourism in a different manner with respect to other forms of rural tourism and constitutes an *unicum* on the international scene (Santucci 2013). In fact, agritourism can only be performed by the farmer and his family members (Law no. 96/2006). Moreover, “the agricultural activity of the farm and not its tourism activities, must be predominant” (Sidali 2011). This predominance of agricultural activity is fixed in terms of working hours and not in terms of income. In other words, it “forces” the agritourism entrepreneur to dedicate himself mainly to agricultural practices. In this sense, the agritourism can be considered a mix of agriculture and tourism.

In summary, agritourism takes up an exclusively Italian characteristic within the European rural tourism scene due to the particular regulatory legislation that plays a key role in agritourism from three points of view: economic, social and environmental.

Theoretically, the organizational model of agritourism is consistent with the environmental sustainability paradigm (Mastronardi and Cipollina 2009) because agritourism leads to an optimal level of external effects (pollution) on a social level, that is Pareto-efficient. This occurs because in Italian legislation, agritourism is an activity “connected” to agriculture and this condition guarantees the concurrence between the benefits of agricultural and tourism activity, at least on a farm level (Belletti 2010).

In Italy, agritourism is already a consolidated phenomenon and represents the most radical product innovation that has ever concerned national agriculture (Esposti 2012, 2006). In 2011, the farms authorized to carry out agritourism activities were slightly more than 20,000 units (about 2 % of the total) (ISTAT 2012). Amongst the services offered, overnight stays are the most important, as in the rest of Europe, followed by food service and product tasting. 20 % of the agritourism farms only offer lodging, while 36 % combine overnight stays and food service and 50 % of them offer, together with lodging, at least one service such as horseback riding, hiking, naturalistic observation, sports (mountain biking, trekking), didactic activities. The agritourism farms that offer other activities, whether or not there is lodging, represent 59 % of the total (ISTAT 2012).

In this context, this paper sheds light on the environmental performances of Italian agritourism farms, which we refer to as agritourisms.

## Methodology

**Fig. 1:**
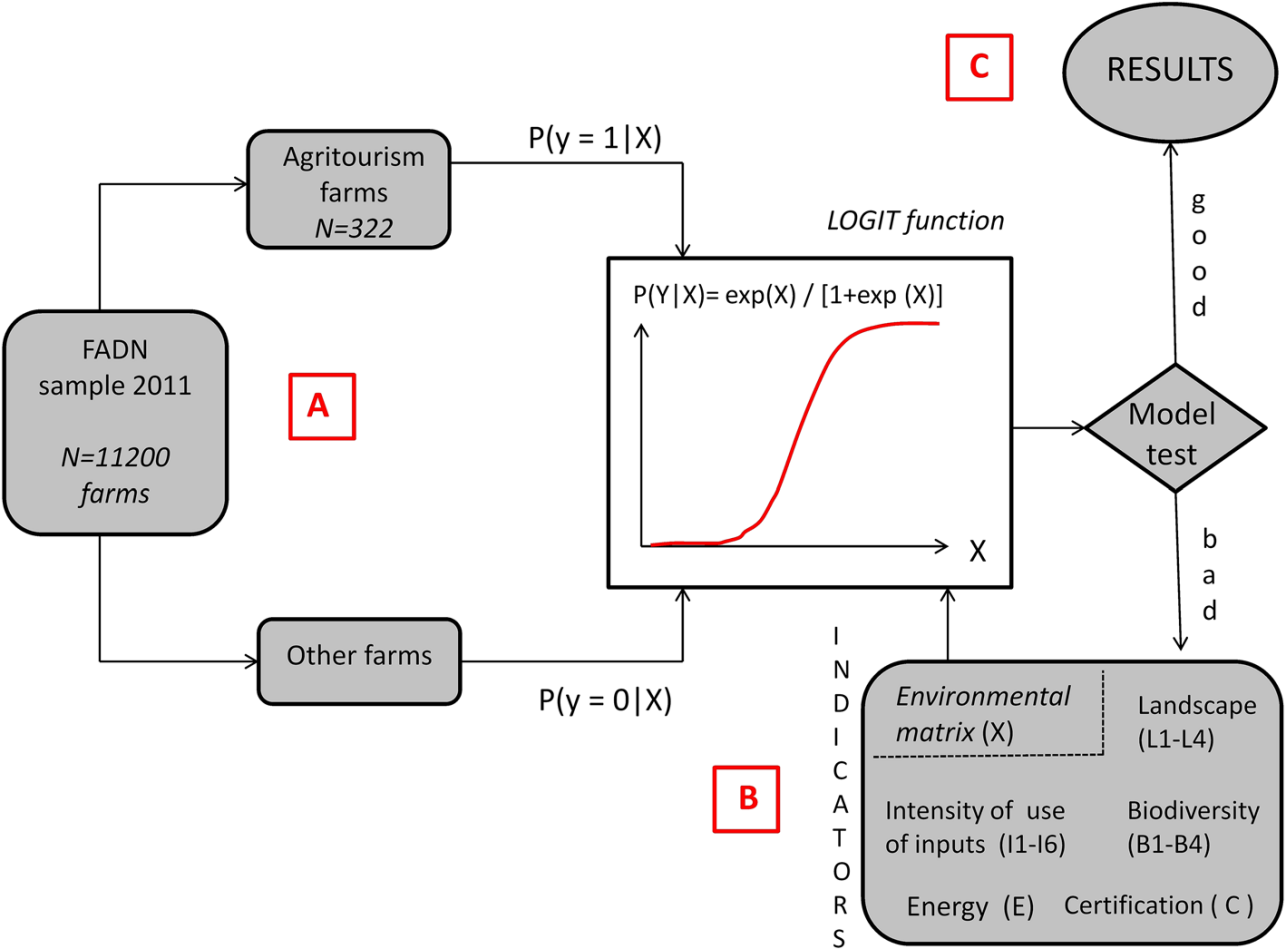
The methodology can be outlined through a flow-chart which describes the main logical steps followed in the discussion. At the beginning (**a**) our sample was divided in two subsamples, one formed by the farms with agritourism and the other by the farms without agritourism. On the second hand, the data analysis was performed both on national sample and on sample sections according to the altitude of the farms. The first sample matches the ones (Y = 1) in our dependent variable and the other matches the zeros (Y = 0). The dichotomous structure of the dependent variable requires a binomial *logit* model (**b**) to be associated to the matrix of explicative or independent variables. This matrix was built by selecting a set of environmental indicators as reported in detail in Additional file 1: Table S1. At last (**c**) the tests applied to assess the fitness of the model may give good or bad results, according to the more or less explicative power of the variables included in the matrix, therefore some of them may be excluded or not from the model

The overall methodology is graphically explained in Fig. 1, which illustrates a flow-chart diagram of the key steps carried out in the model building. The reference sample for data analysis is the FADN sample as described in phar. 2.1.

### Data sample

**Fig. 2:**
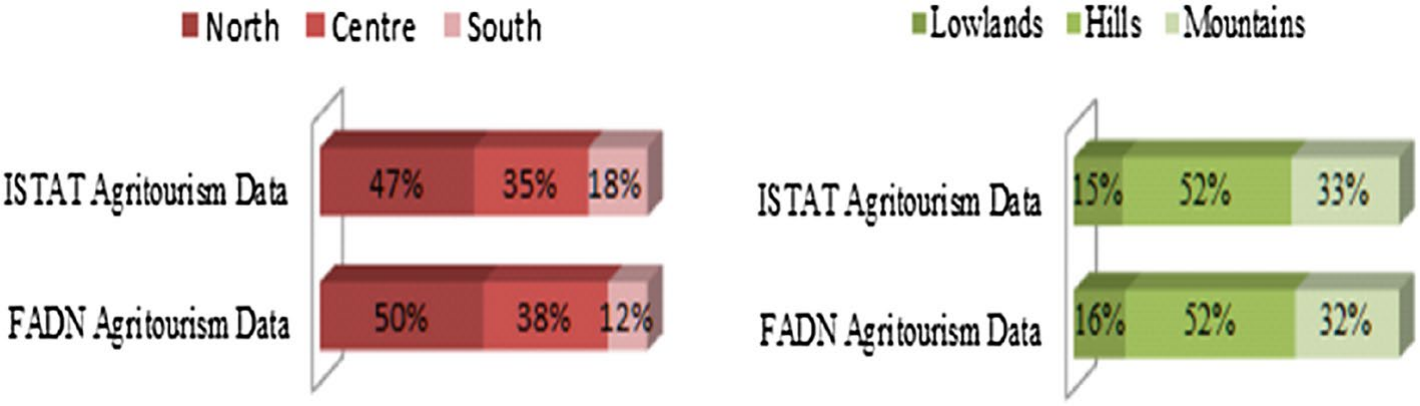
Geographic and altitude distribution of the agritourisms in FADN (Farm Accountancy Data Network) and in the ISTAT (Italian National Institute of Statistics) survey. *Source: our processing of ISTAT and FADN data*

The farm panel analysed is made up of slightly more than 11,200 farms, accounted for in 2011; 372 of these practiced agritourism along with ordinary agricultural practices of cultivation and/or livestock farming. As is known, the FADN (Farm Accountancy Data Network) sample was created to represent the technical and economic operation of the farms in the European Union and certainly not to study the behavior of certain economic sectors, including agritourism that, therefore, cannot be described with statistical rigor by the accounting network. However, without having pretensions to statistically representing the agritourism sector in Italy, the sufficient diffusion within the FADN sample and, above all, a distribution of the FADN agritourisms altogether analogous to the national panorama (Fig. 2), allows some interesting considerations to be made regarding the environmental performances of the activities tied to tourism in rural Italian areas.

The territorial distribution of the entire FADN sample shows that 44 % of the agritourisms are located in the northern regions, while 23 % are located in central Italy and 33 % in the south. The distribution of the quota of agritourisms seems much less balanced; they dominate in the north (50 %) and in central Italy (38 %), while the quota of FADN agritourisms in the south is extremely limited: just 12 %. Moreover, this closely reflects the same distribution of the world of agritourisms observed by ISTAT, the Italian national Institute of Statistics.

Also in terms of the altitude localization, the FADN sample is very similar to the picture highlighted by ISTAT: more than half of the agritourisms are found in hilly areas (52 % in both surveys) and about 1/3 are located in the mountains, while the quota located in the lowlands is marginal (15 % in the survey on agritourisms and 16 % in the FADN survey). In relation to the production systems, agritourism seems to be the most common amongst non-specialized farms (19 %, compared to 11 % of the whole sample), where it is easier to diversify the functions of said production systems. Meanwhile amongst specialized farms, the practice of agritourism is more common in those with arboreal crops and herbivore livestock farming.

### Data processing

The beginning step (Fig. 1a) goes through the comparison of environmental performances of farms with and without agritourism activities. The analysis of the data was carried out on two levels: (a) national, regarding the entire national sample of the FADN Data Bank, and (b) by altitude zones, distinguishing between lowlands, coastal hills, inner hills and mountains. The groups of farms were examined according to the following criteria: (1) landscape conservation; (2) biodiversity conservation; (3) production of energy by alternative sources; (4) adoption of certification systems; and (5) intensity of use of inputs.

The environmental matrix (Fig. 1b) was built by choosing the most frequent indicators (Additional file 1: Table S1) used in studies regarding sustainability (EEA 1998; EC 2001; INEA 2004; OECD 2001a, 2001b; Wascher 2000). The selected variables permit the phenomenon to be measured, in relation to the goals set.

Landscape conservation is evaluated by considering the indicators from L1 to L4 in Additional file 1: Table S1. Crop diversity is expressed by the index of *evenness*, which is a measure of the degree of diversity in a mosaic (landscape or cultivation composition in this specific case), and derives from the Shannon–Weaver Index expressed by ${\text{H }} = \, - \, \sum^{\text{i}} {\text{p}}_{\text{i}} *{\text{ ln p}}_{\text{i}} ,{\text{ for i }} = { 1}, 2, \ldots {\text{N}}$, where p_i_ is the fraction occupied by each type of cultivation and N is the number of types. E*venness* expresses a normalized measurement of the Shannon’s Index of diversity and is given by ${\text{J }} = {\text{ H}}/{\text{H}}_{ \hbox{max} }$, with ${\text{H}}_{ \hbox{max} } = {\text{ ln N}}$. It varies from 0 (only one type present) to 1 (perfect evenness between types, with p_i_ = 1/N).

Biodiversity conservation (indicators from B1 to B4) is represented by the number of plant and animal varieties, by the biological surface area and the farmland falling in protected areas. The plant and animal variety indicates the size relative to a set (*richness*) and is expressed by the Margalef Index given by M = (N − 1) ln C, where N is the number of types (*cultivar* or animal breeds) present and C a measurement of the set, such as the cultivated surface area or the number of heads raised, expressed in Livestock Unit (LU).

The indicator which refers to the production of energy by alternative sources (E) expresses the quota of energy produced by renewable sources present within the farm, while the indicator which regards to the certification systems (C) indicates the number of non-conventional certificates, which the farm uses, according to type and purpose.

Indicators from I1 to I6 highlight the intensity of use of inputs: in particular, I6 expresses the quantity of plant protection products associated with its degree of toxicity and the quantity of nitrogen used in food processing. The degree of phytotoxicity is expressed by $\left( {{\text{P}}_{\text{i}} {\text{Q}}_{\text{i}} } \right)/\sum {\text{ P}}_{\text{i}} {\text{Q}}_{\text{i}} *{\text{ UAA}}$, where Q indicates the quantities of plant protection products and P the weight associated with the class of toxicity, with P = 3 for class I, P = 2 for class II, P = 1 for classes III-IV, and P = 0 for non-toxic plant protection products.

### The model

Regarding the choice of the analytical model (Fig. 1c), the classical linear regression models are not suitable for estimating the value of the dependent variable when there are only two possible cases (presence or absence of agritourism annexed to farms), because the solution does not admit continuous values and error terms are not normally distributed.

The dichotomous structure of the dependent variable (presence or absence of agritourism) results in a propensity towards a method of estimation based on the Binary Response Model Regression (BRMR) (Davidson and MacKinnon 2004).

In the class of generalized linear models, Logit models (Czepiel 2002) equate a given linear combination of independent variables to the probability of observing a unit value in a binary dependent variable, where the value j = 1 expresses the probability of success (presence of agritourism) compared to the alternative j = 0, which identifies the absence of farms with agritourism.

In formal terms, we have

1 $${ \log }[\left( {\pi_{\text{i}} / \, \left( { 1- \, \pi_{\text{i}} } \right)} \right] \, = \, \sum^{\text{k}} {\text{x}}_{\text{ik}} \beta_{\text{k}} ,\quad{\text{ with i }} = { 1}, 2, \ldots ..{\text{N}}$$

where k is the number of independent or explanatory variables listed in Table 1 and $\pi_{\text{i}} = {\text{ P }}\left( {{\text{Zi }} = { 1}|{\text{ i}}} \right)$ is the probability that the random variable ${\text{Z }} = \, \left( {{\text{z}}_{ 1} ,{\text{ z}}_{ 2} , \, \ldots .{\text{z}}_{\text{N}} } \right)$ associated with the N observations of examined sample (FADN) takes a unit value (success) for the ith case.

The Eq. (1) is equal to:

2 $${\text{P }}\left( {{\text{y}}_{\text{i}} = { 1}|{\text{ x}}_{\text{i}} } \right) \, = \Lambda \left( {\text{X}} \right) \, = {\text{ e}}^{\text{X}} / \left( { 1 { } + {\text{ e}}^{\text{X}} } \right)$$

where Λ (X) is the cumulative distribution function (CDF) of a standardized logistic distribution and X is the matrix of explanatory variables.

The purpose of the logistic transformation is to estimate the k beta parameters of the Eq. (1), and this may be done using a maximum likelihood function like the following one:

3 $${\text{L }}\left( {\beta |{\text{y}}} \right) \, = \, \varPi^{\text{N}} {\text{n}}_{\text{i}} !/{\text{y}}_{\text{i}} !\left( {{\text{n}}_{\text{i}} - {\text{ y}}_{\text{i}} } \right)! \, \left[ {\pi_{\text{i}}^{\text{yi}} \left( { 1 { }{-} \, \pi_{\text{i}} } \right)^{{{\text{ni}}\;{ - }\;{\text{yi}}}} } \right]$$

The formula (3) comes from the probability distribution of the dependent variable, which is a joint density function of binomial type, and is equivalent to the parametric estimate of the function that maximizes the probability of observing the sample ${\text{z }} = \, \left( {{\text{z}}_{ 1} ,{\text{ z}}_{ 2} , \, \ldots {\text{ z}}_{\text{N}} } \right)$.

The conditions to be fulfilled for the estimation are expressed by (4) and (5):

4 $${\text{dL }}\left( {\beta \, |{\text{ y}}} \right)/{\text{ d}}\beta = 0$$

5 $${\text{dL }}\left( {\beta \, |{\text{ y}}} \right)/{\text{ d}}^{ 2} \beta \, < \, 0$$

Because of the high complexity of the estimation method, the use of algorithms implemented in specialized software is required: in our study GRETL econometric software was used for this purpose (Cottrell and Lucchetti 2012), selecting the ‘robust standard errors’ option in order to get more significant results: in fact, this option provides consistent estimates on average, even in case of autocorrelation between the error terms, and proves to be particularly useful for large datasets that are not suitable to traditional estimation methods.

Tests regarding the goodness of fit of the Logit model (likelihood relationship) and tests relative to the estimation capacity of the model (presence/absence of an agritourism connected to the farm) were carried out (Additional file 2: Table S2; Fig. 1c) for each sample group. Firstly, the global significance of the model is expressed by the variable Chi squared test (χ^2^) whose values increase when the probability of the null hypothesis decreases^1^; secondly, the number of cases estimated by the model is compared, through confusion matrixes, with the total number of cases observed for both values (0 and 1) in the dependent variable, taking on the average of the dependent variable as the threshold value.

Model tests show an elevated significance of the model [*p* value <10^−4^] and an overall predictive capacity greater than 90 % of the reference mark used. However, the low representativeness of the agritourisms on the total number of farms examined shows that most of the correct predictions are referred to the most numerous class (farms without agritourism), while the class with agritourisms, which counts about 3 % out of total observations, is affected by a higher prediction error.

## Results

In agreement with the initial hypothesis, the results show, overall, that agritourisms pay greater attention to the aspects regarding environmental matter as compared to the other farms on the panel. This tendency emerges from the values of the different indicators as regards the conservation of ecosystems and landscape, biodiversity, the production of energy by renewable sources, by the adoption of certification systems and intensity of use of inputs. In this regard, Additional file 1: Table S1 illustrates the associations between the agritourisms and the indicators of sustainability taken into consideration for the entire national territory (Full sample). For each indicator, Additional file 3: Table S3 shows the coefficients estimated by the model and the marginal contributions, calculated as an average value of the marginal effects^2^. The marginal contributions express the connected variations of the probability of observation of the dependent variable for each unitary variation of the independent variables and therefore the relative importance of the variable. The sign of the coefficient indicates the predominant direction of the association: with the positive sign (+) for direct association, with the negative sign (−) for the inverse association. For analysis of the results, only the indicators with confidence intervals greater than 90 % (p < 0.1) were used.

The application of the Logit model on a national scale shows, firstly, that the agritourisms have, on average, a greater variety of animals raised (B2) and use a greater number of renewable sources (E) as compared to farms without agritourism. Greater variety of animals raised is very important for biodiversity conservation because animal genetic resources and animal management systems are an integral part of ecosystems and productive landscapes in Italy. Traditional production systems required multipurpose animals, which, although less productive than high-output breeds, may contain valuable functional traits. There are breeds that are interesting because they exhibit a desirable trait or bear a gene pool with potential use. Although the local breeds are usually not competitive for production traits, they may carry valuable features such as disease resistance or distinctive product quality. The production of energy from renewable sources makes both economic and environmental benefits. They contribute to reducing energy dependency and counteracting climate change. In agriculture, production of renewable energy is a possibility of integration of farmers’ income and an opportunity to diversify productive activities. Renewable energy technologies utilization is indicated as an appropriate alternative for providing a considerable portion of future energy demand. Renewable energy has the potential to play an important role in providing energy with sustainability to the vast populations.

This confirms a broadening of activities beyond traditional ones, which is characterized more and more in a multifunctional sense. Secondly, agritourisms are characterized by a more controlled use of soil expressed by the minimum UAA/TAA ratio (I4) and, consequentially, by a greater presence of forest surface area (L3). This may suggest a higher incidence of woodlands, natural and semi-natural areas in the farming systems, confirming the effectiveness of agri-environmental policies related to the conservation of high natural value. The agritourisms show a lesser intensity of use of inputs as to water resources (I1 and I2), which expresses a more controlled pressure on natural resources.

It may be useful to remember that agriculture is often accused of creating significant harm to water resources, both from a quantitative point of view, through the continuous withdrawals, both in terms of quality, contributing to the pollution of surface waters, groundwater and soil. The use of water for productive purposes can generate negative effects on the environment that result in depletion of the aquifer, increased soil erosion, salinization of soils, groundwater contamination from minerals and the reduction of wetlands, which causes, as a consequence, the destruction of natural habitats. Therefore agritourisms, by increasing the efficiency of irrigation practices, help actively to solve the described problems, both from a technical and managerial point of view, as well by choosing cropping systems better suited to the characteristics of the different agro-climatic areas. Lastly, the inclination towards adoption of certification systems of a biological type and regarding the origin of the products (C) are significant, evidencing positive effects on biodiversity and food quality with a low intake level. Organic farming can reduce the negative effects of intensive agriculture and may offer a possible answer to the concerns about the environmental impact and the efficiency of primary sector.

The analysis of the agritourisms broken down by altitude areas (lowlands, coastal hills, inner hills, and mountains; Additional file 4: Table S4) confirms the majority of these tendencies, but it also highlights others that did not emerge in the global analysis.

The agritourisms located in lowlands present differences analogous to the national situation as to the zootechnical variety (B2), which represents the most relevant positive contribution, and production of energy by renewable sources (E). The most important difference with respect to the national context regards the significantly lower consumption of fertilizers in irrigation per unit of surface area (I3) with positive effects on ecosystems, soils and human health. This is probably associated in part with the most reduced irrigation surface areas (I1) and with tourism demand, which focuses on quality food goods and an increasing interest in the principles of organic and ecological agriculture. The second difference in terms of importance regards the low incidence of agritourisms falling in protected areas (B4). A possible explanation for this phenomenon is the representativeness of lowland ecosystems in the Italian panorama of protected areas that have greater tourism attractiveness. Most of these environments are coastal areas, which are, on average, distant from the agritourisms generally situated in nearby inland areas. The other differences are observed in the greater use of low-power agricultural machinery (I5) with positive effects on the soil, and the presence of more diversified landscapes (L1) with a greater presence of arboreal crops (above all grapevines and olive trees), probably to meet the requests for variety made by tourists and to transmit food to consumers for final consumption. The landscape quality is the necessary basis for productive activities, such as agriculture and tourism. Agritourism is an inherently territorial activity, really connected to the specific territory, in terms of environmental diversity, architectural texture, cultural and social wealth. Although relevant, but not very significant, is the consistency of meadow and pasture farm surface areas (L2), which confirms the effectiveness of agri-environmental policies relative to the conservation of semi-natural areas in territories that present phenomena of environmental impact due to intensive farming.

Amongst agritourisms located in the coastal hills, the only relevant result indicated is represented by the production of renewable energy (E). This aspect is found with the same and high level of significance in the remaining altitude areas, which confirms the centrality of the role of energy in terms of strategies directed toward natural resources conservation.

In the inner hills, the most significant variables are the greater species diversity of animal breeds (B2), the highest quota of energy produced by renewable sources (E) and, above all, the marked reduction of fertirrigated surface areas (I3). Because of such characteristics, the agritourisms in this area present similarities with those located in the lowlands. They are slightly different from the latter since they have greater surface areas used for non-agricultural purposes (L4), which indicates a positive effect for the relationship between farming and environmental conservation. Considering the fact that the continuation of agricultural productive processes is positive for the landscape and the biodiversity in said areas, this aspect is reinforced by the consistency of forest surface areas (L3) that prevails over meadows and pastures (L2).

The agritourisms located in mountain areas are also characterized by a lesser use of fertirrigation (I3), by the lowest UAA/TAA ratio (I4) and, in particular, by the lowest quantity and level of dangerousness of the plant protection products used (I6), with respect to farms located in the same area, although this aspect is much less relevant in terms of contribution and significance compared to the previous ones.

## Conclusions

This study has clarified the relationship between the Italian agritourisms and the environment and highlighted how the agritourism is characterized as a productive activity with the least environmental impact in the Italian agricultural panorama. Farms with agritourisms tend to develop more sustainable techniques that have a positive impact on biodiversity, landscape and on natural resources. In this manner, agritourisms represent an opportunity to reduce the negative external effects of agriculture on the environment, even though their performances, from an economic and social point of view, are probably inferior to other companies operating in rural areas (Colton and Bissix 2005).

The analysis carried out in this paper provides the means to reflect upon the very real possibility that farm-based agritourism promotes the spreading of the most sustainable production models. If this is indeed the case, farm-based agritourism also promotes the most effective policies to support these initiatives, or, on the contrary, upon those that are most useful in strengthening this aspect.

The environmental performances of agritourisms can be seen as the result of a farm diversification process aimed towards the development of environment-based services (Barbieri 2013). This is possible because Italian law has considered agritourism as a support activity of the farm and never dominant with respect to traditional farming. In other words, said legislation has compelled the farmer to consider the offer of tourism services as secondary to the primary agricultural activities, even though these services are usually more lucrative and, above all, characterized by a quick economic return. In a way, the remuneration deriving from their offer of services and tourism activities is a reward for the activities aiming toward the conservation of biodiversity, the guarantee of ecosystem services and those regarding landscape preservation. Additionally, one must not forget that said elements usually represent the *leitmotif* of tourism destinations (Franch 2010; Deuffic and Candau 2006). The idea is that tourism in the farm can become the redemptive economic activity of rural areas involved in the phenomena of marginality, with positive implications for the natural resources of the territories concerned.

In this regard, the policies focused on agritourism can be considered the most effective in terms of sustainability. They manage to achieve two objectives: promoting rural development and protecting the environment. These policies also deal with the problems of efficiency and practicality involving complex inter-related topics of economic, social and environmental policies.

In conclusion, the data as analysed in this study suggests that the policies underlying agrotourism in Italy are achieving their goals.

## Additional files

10.1186/s40064-015-1353-4 Indicator’s description.
10.1186/s40064-015-1353-4 Model Estimation Test.
10.1186/s40064-015-1353-4 Estimation of the Binomial Logit Model - Full sample.
10.1186/s40064-015-1353-4 Estimation of the Binomial Logit Model – Altitude areas (Lowlands, Coastal Hills, Inner Hills, Mountains).

## Footnotes

1 Shown in parenthesis […] until 4 significant figures.

2 In a non-linear model such as Logit, the marginal effects of the variables are not constant but vary according to the value assumed by the whole of the explanatory variables. For this reason, a correct estimate calls for the calculation of the average value of the derivative of the Logit function: d Λ(X)/dx = d [exp X)/[1 + exp X)]/dx = exp (X)/[1 + exp (X)]^2^.
