# Supplementary material for: Is agritourism eco-friendly? A comparison between agritourisms and other farms in Italy using farm accountancy data network dataset
Source: Springerplus. 2015 Oct 12;4:590. doi: 10.1186/s40064-015-1353-4 (PMC4627998; doi:10.1186/s40064-015-1353-4)
Supplement: Supplementary file 1 — 10.1186/s40064-015-1353-4 Indicator’s description. [file 40064_2015_1353_MOESM1_ESM.doc]

Table S1: Indicator’s description

| **Fields** | **Indicators** | **Description** |
| --- | --- | --- |
| Landscape | L1 | Crop diversification |
|  | L2 | % surface area of meadows and pastures |
|  | L3 | % forest surface area |
|  | L4 | % non-UAA surface area |
| Biodiversity | B1 | Plant variety |
|  | B2 | Animal variety |
|  | B3 | % Biological surface area |
|  | B4 | % farm land falling in protected area |
| Energy | E | % energy produced by renewable sources |
| Certification | C | Number of certificates by type and purpose |
| Intensity of use of inputs | I1 | % irrigated surface area |
|  | I2 | Volume of water distributed (hl/ hc) |
|  | I3 | % fertirrigated surface area |
|  | I4 | UAA/TAA ratio[[1]](#footnote-2) |
|  | I5 | Horse-power (Kw/UAA) |
|  | I6 | Yearly average quantity of phytopharmaceuticals (quintals) per hectare per plant species, weighted with the class of toxicity |

1. Utilised Agricultural Area (UAA) on Total Agricultural Area (TAA). [↑](#footnote-ref-2)
