# Supplementary material for: Is agritourism eco-friendly? A comparison between agritourisms and other farms in Italy using farm accountancy data network dataset
Source: Springerplus. 2015 Oct 12;4:590. doi: 10.1186/s40064-015-1353-4 (PMC4627998; doi:10.1186/s40064-015-1353-4)
Supplement: Supplementary file 2 — 10.1186/s40064-015-1353-4 Model Estimation Test. [file 40064_2015_1353_MOESM2_ESM.docx]

Table S2: Model Estimation Test

| ITALY | | Likelihood relationship  χ^2^(17) = 805,611 [0,0000] | | | | Plains | | Likelihood relationship  χ^2^(17) = 183,918 [0,0000] | | | | Costal  hills | | Likelihood relationship  χ^2^(17) = 88,2285 [0,0000] | | | |
| --- | --- | --- | --- | --- | --- | --- | --- | --- | --- | --- | --- | --- | --- | --- | --- | --- | --- |
|  |  | **Effective** | | Total sample | %  correct |  |  | **Effective** | | Total sample | %  correct |  |  | **Effective** | | Total sample | %  correct |
|  |  | **0** | **1** |  |  |  |  | **0** | **1** |  |  |  |  | **0** | **1** |  |  |
| **Expected** | **0** | 10823 | 41 | 10864 | 99.6 | **Expected** | **0** | 3795 | 5 | 3800 | 99.9 | **Expected** | **0** | 1586 | 6 | 1592 | 99.6 |
|  | **1** | 309 | 65 | 374 | 17.4 |  | **1** | 54 | 7 | 61 | 11.5 |  | **1** | 28 | 10 | 38 | 26.3 |
| Total correct = 10888 | | | | 11238 | 96.9 | Total correct = 3802 | | | | 3861 | 98.5 | Total correct =1596 | | | | 1630 | 97.9 |
| **Average value of dependent variable:**  ITALY: 0.033280  Lowlands: 0.015799  Coastal hills: 0.023313  Inner hills: 0.044489  Mountains: 0.053201 | | | | | | Inner  hills | | Likelihood relationship  χ^2^(17) = 379,936 [0,0000] | | | | Mountains | | Likelihood relationship  χ^2^(17) = 192,029 [0,0000] | | | |
|  |  |  |  |  |  |  |  | **Effective** | | Total sample | %  correct |  |  | **Effective** | | Total sample | %  correct |
|  |  |  |  |  |  |  |  | **0** | **1** |  |  |  |  | **0** | **1** |  |  |
|  |  |  |  |  |  | **Expected** | **0** | 3340 | 32 | 3372 | 99.1 | **Expected** | **0** | 2083 | 17 | 2100 | 99.2 |
|  |  |  |  |  |  |  | **1** | 103 | 54 | 157 | 34.4 |  | **1** | 93 | 25 | 118 | 21.2 |
|  |  |  |  |  |  | Total correct = 3394 | | | | 3529 | 96.2 | Total correct =2108 | | | | 2218 | 95.0 |
