# Supplementary material for: Is agritourism eco-friendly? A comparison between agritourisms and other farms in Italy using farm accountancy data network dataset
Source: Springerplus. 2015 Oct 12;4:590. doi: 10.1186/s40064-015-1353-4 (PMC4627998; doi:10.1186/s40064-015-1353-4)
Supplement: Supplementary file 3 — 10.1186/s40064-015-1353-4 Estimation of the Binomial Logit Model - Full sample. [file 40064_2015_1353_MOESM3_ESM.doc]

Table S3: Estimation of the Binomial Logit Model - Full sample

| **Variables** | **Coefficient**  **(SE)** | **MME** | **SL** |
| --- | --- | --- | --- |
| **L1** | -0.1777  (0.1829) | -0.0397 |  |
| **L2** | 0.3302  (0.2039) | 0.0808 |  |
| **L3** | 1.2093  (0.6415) | 0.3010 | * |
| **L4** | 0.1189  (0.1090) | 0.0294 |  |
| **B1** | 0.2058  (0.3829) | 0.0509 |  |
| **B2** | 4.5007  (0.8961) | 11.245 | *** |
| **B3** | 0.5145  (0.3202) | 0.1279 |  |
| **B4** | -0.0275  (0.1410) | -0.0069 |  |
| **E** | 3.7128  (0.1508) | 0.9232 | *** |
| **C** | 0.1060  0.0358) | 0.0229 | *** |
| **I1** | -1.0180  (0.3629) | -0.2505 | *** |
| **I2** | -0.0009  (0.0004) | -0.0002 | ** |
| **I3** | -0.6076  (1.2071) | -0.1515 |  |
| **I4** | -1.4480  (0.6383) | -0.2976 | ** |
| **I5** | 0.0113  (0.1935) | 0.0028 |  |
| **I6** | -0.0100  (0.0130) | -0.0024 |  |
| **Constant** | −2.8110  (0.5949) | 0.0000 | *** |

MME = Medium Marginal Effects; (SE) = Standard Errors; SL = Significance Level [ (***): p <0.01; (**): p <0.05; (*): p <0.10 ]
