# Supplementary material for: Is agritourism eco-friendly? A comparison between agritourisms and other farms in Italy using farm accountancy data network dataset
Source: Springerplus. 2015 Oct 12;4:590. doi: 10.1186/s40064-015-1353-4 (PMC4627998; doi:10.1186/s40064-015-1353-4)
Supplement: Supplementary file 4 — 10.1186/s40064-015-1353-4 Estimation of the Binomial Logit Model – Altitude areas (Lowlands, Coastal Hills, Inner Hills, Mountains). [file 40064_2015_1353_MOESM4_ESM.doc]

Table S4: Estimation of the Binomial Logit Model – Altitude areas (Lowlands, Coastal Hills, Inner Hills, Mountains)

|  | **Lowlands** | | | | | **Coastal Hills** | | | | **Inner Hills** | | | | **Mountains** | | | |
| --- | --- | --- | --- | --- | --- | --- | --- | --- | --- | --- | --- | --- | --- | --- | --- | --- | --- |
| **Variables** | **Coefficient**  **(SE)** | | **MME** | | **SL** | **Coefficient**  **(SE)** | | **MME** | **SL** | **Coefficient**  **(SE)** |  | **MME** | **SL** | **Coefficient**  **(SE)** |  | **MME** | **SL** |
| **L1** | 1.1515  (0.4470) |  | | 0.2555 | *** | 0.5571  (0.6087) |  | 0.1239 |  | 0.4546  (0.3560) |  | 0.1010 |  | - 0.2149  (0.2985) |  | -0.0492 |  |
| **L2** | 1.3058  (0.6922) |  | | 0.3249 | * | 0.0578  (0.8833) |  | 0.0143 |  | - 1.3841  0.5314) |  | -0.3413 | *** | 0.3169  (0.2658) |  | 0.0739 |  |
| **L3** | - 0.3797  (1.8443) |  | | -0.0948 |  | 1.0392  (1.3249) |  | 0.2593 |  | 2.7169  (1.3700) |  | 0.6756 | ** | - 0.3328  (0.9887) |  | -0.0823 |  |
| **L4** | - 2.5498  (7.0394) |  | | -0.6344 |  | 0.0555  (0.5701) |  | 0.0138 |  | 1.1002  (0.4806) |  | 0.2719 | ** | - 0.8274  (1.0045) |  | -0.2037 |  |
| **B1** | - 0.6491  (1.2637) |  | | -0.1603 |  | 1.1497  (1.0828) |  | 0.2848 |  | 0.4495  (0.6484) |  | 0.1110 |  | 0.4355  (0.5742) |  | 0.1078 |  |
| **B2** | 12.810  (3.2832) |  | | 3.2021 | *** | 3.8588  (3.4201) |  | 0.9643 |  | 4.6033  (1.1643) |  | 1.1499 | *** | 0.7535  (1.4844) |  | 0.1881 |  |
| **B3** | - 0.2157  (0.8282) |  | | -0.0537 |  | 1.0173  (0.8448) |  | 0.2518 |  | 0.3936  (0.4417) |  | 0.0977 |  | 0.5719  (0.6451) |  | 0.1422 |  |
| **B4** | - 6.3533  (1.7833) |  | | -1.5859 | *** | 0.5940  (0.5671) |  | 0.1478 |  | - 0.3530  (0.7733) |  | -0.0879 |  | 0.3482  (0.6327) |  | 0.0866 |  |
| **E** | 3.7258  (0.3549) |  | | 0.9268 | *** | 4.7261  (0.6156) |  | 1.1786 | *** | 4.0919  (0.2494) |  | 1.0162 | *** | 3.4177  (0.3373) |  | 0.8492 | *** |
| **C** | 0.2317  (0.0616) |  | | 0.0527 | *** | 0.0424  (0.0873) |  | 0.0090 |  | 0.1182  (0.0437) |  | 0.0247 | *** | 0.0373  (0.0678) |  | 0.0079 |  |
| **I1** | - 3.9230  (1.6406) |  | | -0.9549 | ** | - 0.0705  (1.0408) |  | -0.0174 |  | - 1.2883  (0.9678) |  | -0.3193 |  | - 0.4146  (0.5937) |  | -0.1026 |  |
| **I2** | - 0.0012  (0.0003) |  | | -0.0003 | *** | 0.0038  (0.0164) |  | 0.0009 |  | 0.0148  (0.0139) |  | 0.0036 |  | 0.0084  (0.0084) |  | 0.0020 |  |
| **I3** | -78.677  (11.172) |  | | -19.5809 | *** | 1.4129  (1.8714) |  | 0.3526 |  | - 105.86  (10.318) |  | -26.419 | *** | - 29.148  (2.4207) |  | -7.2698 | *** |
| **I4** | - 4.8171  (7.1445) |  | | -0.9786 |  | - 0.7881  (1.2145) |  | 0.1626 |  | 1.5702  (1.4554) |  | 0.3240.3240 |  | - 3.4999  (1.4184) |  | -0.7276 | ** |
| **I5** | - 1.0777  (0.5434) |  | | -0.2658 | ** | 0.3508  (0.3466) |  | 0.0853 |  | - 0.2746  (0.5633) |  | -0.0680 |  | 0.2135  (0.2896) |  | 0.0525 |  |
| **I6** | - 0.0390  (0.0572) |  | | -0.0078 |  | - 0.0844  (0.0897) |  | -0.0184 |  | 0.0093  (0.0079) |  | 0.0019 |  | - 0.2150  (0.1117) |  | -0.0529 | * |
| **Constant** | 0.6729  (7.1192) |  | |  |  | - 4.2824  (1.2486) |  |  | *** | - 5.7987  (1.4449) |  |  | *** | - 0.2573  (1.3482) |  |  |  |

MME = Medium Marginal Effects; (SE) = Standard Errors; SL = Significance Level [ (***): p <0.01; (**): p <0.05; (*): p <0.10 ]
